# Comparison of Prioritisation Schemes for Human Pharmaceuticals in the Aquatic Environment

**DOI:** 10.1007/s11356-018-3834-9

**Published:** 2018-12-04

**Authors:** Sarah Letsinger, Paul Kay

**Affiliations:** School of Geography, University of Leeds, Woodhouse Lane, Leeds, West Yorkshire LS2 9JT UK

**Keywords:** Prioritisation, Pharmaceuticals, PEC, FPM, Toxicity, Ranking

## Abstract

Only a small proportion of pharmaceuticals available for commercial use have been monitored in the aquatic environment, and even less is known about the effects on organisms. With thousands of pharmaceuticals in use, it is not feasible to monitor or assess the effects of all of these compounds. Prioritisation schemes allow the ranking of pharmaceuticals based on their potential as environmental contaminants, allowing resources to be appropriately used on those which are most likely to enter the environment and cause greatest harm. Many different types of prioritisation schemes exist in the literature and those utilising predicted environmental concentrations (PECs), the fish plasma model (FPM), critical environmental concentrations (CECs) and acute ecotoxicological data were assessed in the current study using the 50 most prescribed drugs in the UK. PECs were found to be overestimates of mean measured environmental concentrations but mainly underestimations of maximum concentrations. Acute ecological data identified different compounds of concern to the other effects assessments although the FPM and CECs methods were more conservative. These schemes highlighted antidepressants, lipid regulators, antibiotics, antihypertensive compounds and ibuprofen as priority compounds for further study and regulation.

**Electronic supplementary material:**

The online version of this article (10.1007/s11356-018-3834-9) contains supplementary material, which is available to authorized users.

## Introduction

Concern over the presence of pharmaceuticals in the environment and the subsequent development of environmental risk assessments (ERAs) for these compounds began in the 1990s (Küster and Adler 2014). Currently, only Europe and the USA have specific ERA protocols for the assessment of pharmaceuticals, which are required to be completed in order to register them for commercial use (Adler et al. 2008). In 2006, an EU regulation on the registration, evaluation, authorisation and restriction of chemicals (REACH) came into effect, and now all chemicals being manufactured in or imported to the EU must be assessed following ECHA guidelines, including information on potential risks and hazards to the environment. However, prior to the implementation of such legislation, pharmaceuticals have been released into the environment unregulated for years. The number of human pharmaceuticals in use has been reported as being between 1500 and 10,000 (Guo et al. 2016; Dong et al. 2013). Only a little over 200 of these have been monitored in freshwaters and fewer in marine waters, and even less is known about their impacts once they enter the aquatic environment (Fabbri and Franzellitti 2016; Hughes et al. 2013). This has left continuing uncertainty surrounding the environmental impacts of pharmaceuticals in the aquatic environment. The use of a prioritisation scheme can help address this by identifying a smaller set of compounds which have the potential to enter the environment and pose a biological risk. This can allow researchers and policy makers to direct resources towards further study; they can help decide which compounds need to be monitored in the environment and which require more information on their fate and biological effects (Mansour et al. 2016).

Many prioritisation schemes are based on existing ERAs, which include the calculation of predicted environmental concentrations (PECs) and an assessment of the risk to biota. PECs are usually derived from usage data on the volume of drugs produced per year, or number of prescriptions filed, which may be further refined based on processes which affect the compounds between production and entering the environment, such as metabolism, wastewater treatment and dilution (Besse and Garric 2008). Often, where experimental data is missing or chemical properties are not known, simplified PECs, where little or no fate criteria are applied, may be calculated to facilitate quick assessment of a large number of chemicals (Ashton et al. 2004; Besse and Garric 2008; Kostich and Lazorchak 2008). As a result, the PECs calculated in such schemes give broad predictions for a country or large area and are not refined enough to give predictions at different spatial or temporal scales.

PECs are usually paired with assessments of hazards to biological organisms inhabiting the receiving environments. One such method is through the use of risk quotients, which determine if the predicted no effect concentrations (PNECs) of a compound exceed PECs. If the result is greater than 1 then the study compound is deemed to pose a threat (Hoyett et al. 2016). PNECs are usually calculated by selecting the most sensitive LC_50_ and applying an assessment factor (Backhaus and Faust 2012). Such experimental data is often unavailable in the literature, however, and it is time consuming to generate such data for a prioritisation scheme. Ecotoxicological structure-activity relationships (ECOSAR) can be used to calculate chronic and acute LC_50_ values and are allowed under REACH guidelines (Sanderson et al. 2004; Ortiz de García et al. 2013).

Pharmaceuticals are unique contaminants as they are designed to be biologically active and, unlike many other environmental contaminants, information from the medical literature on the pathways and effects of pharmaceuticals in vertebrates is abundant. This information has been utilised to produce alternative methods of assessing the hazard of pharmaceuticals to biota. Fish are not biochemically different from vertebrates and share many of the same drug targets (Huggett et al. 2003). The fish plasma model utilises this information and compares the human therapeutic concentration to a calculated fish plasma concentration. Vertebrates are usually more sensitive to chemicals than invertebrates, due to shared targets. It is thought that this model is a scheme sufficient to predict the environmental hazard of chemicals (LaLone et al. 2014).

Despite their extensive development, the prioritisation schemes which exist in the literature are varied and often highlight different compounds of concern (Besse and Garric 2008; Donnachie et al. 2016; Roos et al. 2012). Moreover, it can be difficult to compare them as they are applied to different data sets and scenarios which can make it hard to understand which compounds really are of most concern or to select a scheme for use in research and management. The aim of this paper was, therefore, to use a range of common prioritisation schemes to assess the environmental risk of the 50 most prescribed pharmaceuticals in the UK, highlight compounds of concern, and make suggestions as to the efficacy of the different schemes.

## Methods

### Predicted environmental concentrations

#### Calculations

Information on the quantity of pharmaceuticals prescribed was obtained from data released monthly by the National Health Service England for 2014 (NHS 2014). The 50 most prescribed compounds during this period were used for this assessment. For each compound, the monthly and annual mass of prescriptions was calculated (supplementary material S1).

PEC_A_ was calculated using (Eq. 1), where A is the amount of pharmaceuticals dispensed (kg year^−1^), E is the fraction of the compound excreted unchanged, V is the volume of waste water per capita per day (assumed to be 200 l), P is the population of England in 2014, and D is the dilution of waste water (assumed to be 10 times; EMEA 2006). This method was derived from the approach detailed in the EU technical guidance for risk assessment of human pharmaceuticals (EU 2003). Excretion rates were obtained from peer-reviewed literature or online databases and the highest excretion rate was used in the calculation (supplementary material S2). PEC_B_ further refined this equation by applying the removal rate for pharmaceuticals in WWTPs (Eq. 2), where R is the removal rate. Removal rates were obtained from peer-reviewed literature and where multiple removal rates were published for the same compound, the lowest was chosen in order to create a more conservative estimate (supplementary material S2). If no removal rate, or a negative one, was found then it was assumed to be 0. PEC_C_ included further refinement, taking into account metabolism and removal in wastewater (Eq. 3).

1 $$ {\mathrm{PEC}}_{\mathrm{A}}=\frac{\mathrm{A}\times \mathrm{E}}{\mathrm{V}\times \mathrm{P}\times \mathrm{D}\times 365} $$

2 $$ {\mathrm{PEC}}_{\mathrm{B}}=\frac{\mathrm{A}\times \left(1-\mathrm{R}\right)}{\mathrm{V}\times \mathrm{P}\times \mathrm{D}\times 365} $$

3 $$ {\mathrm{PEC}}_{\mathrm{C}}=\frac{\mathrm{A}\times \mathrm{E}\times \left(1-\mathrm{R}\right)}{\mathrm{V}\times \mathrm{P}\times \mathrm{D}\times 365} $$

PEC_D_ (Eq. 4) is derived from the EMEA guidelines and does not require prescription data to be calculated. Instead, it includes the proportion of the population being treated with a particular drug (Fpen), where a suggested value of 1% is used (EMEA 2006). Dose is the maximum dosage per person and Cap_stp_ is the capacity of the local WWTP (assumed to be 10,000; EMEA 2006). The EMEA guidelines also suggest the inclusion of information on the fraction of the compound absorbed to suspended matter. Due to the unavailability of this data for most compounds, this was not included (Besse et al. 2008).

4 $$ {\mathrm{PEC}}_{\mathrm{D}}=\frac{{\mathrm{Elocal}}_{\mathrm{water}}\times \left(1-\mathrm{R}\right)}{\mathrm{V}\times \mathrm{D}\times {\mathrm{Cap}}_{\mathrm{stp}}} $$

5 $$ {\mathrm{Elocal}}_{\mathrm{water}}=\mathrm{Dose}\times \mathrm{E}\times \mathrm{Fpen}\times {\mathrm{Cap}}_{\mathrm{stp}} $$

Each compound was ranked by each of the PEC calculations (supplementary material S3) and the mass prescribed annually in order to compare how the different schemes altered the predicted relative environmental risk.

#### Comparison with environmental concentrations

In order to compare the PECs to measured environmental concentrations (MECs), data were taken from monitoring studies carried out in the UK (Baker and Kasprzyk-Hordern 2013; Bound and Voulvoulis 2006; Burns et al. 2017; Burns et al. 2018a; Kasprzyk-Hordern et al. 2008; Kasprzyk-Hordern et al. 2009; Kay et al. 2016; Nakada et al. 2017; Roberts and Thomas 2006; Ashton et al. 2004). Only monitoring studies from surface water were included, measurements from influent and effluent were omitted. The mean MEC across all studies was calculated and compared to each of the PECs along with the maximum MEC.

### Effect data

#### Fish plasma model

The FPM was calculated according to Huggett et al. (2003). This model compares the human therapeutic plasma concentration (H_T_PC) and the fish steady state concentration (F_ss_PC) to give an effective ratio (ER), a measure of risk (Eq. 6). F_ss_PC was estimated for each of the PEC values calculated in “Calculations” section (Eq. 7) and the H_T_PC was obtained by using the peak serum concentration that is reached in humans after the drug has been administered (cmax). Where multiple cmax values were found, the higher value was used in this assessment (supplementary information S4).

6 $$ \mathrm{ER}=\frac{{\mathrm{H}}_{\mathrm{T}}\mathrm{PC}}{{\mathrm{F}}_{\mathrm{SS}}\mathrm{PC}} $$

7 $$ {\mathrm{F}}_{\mathrm{SS}}\mathrm{PC}=\mathrm{PEC}\kern.3em \mathrm{x}\times {\mathrm{P}}_{\mathrm{Blood}:\mathrm{Water}} $$

8 $$ {\mathrm{logP}}_{\mathrm{Blood}:\mathrm{Water}}=0.73\times {\log}_{\mathrm{kow}} $$

The compounds were ranked from lowest to highest by ER. Huggett et al. (2003) suggested that compounds with an ER < 1000 may warrant further assessment.

#### Critical environmental concentrations

Critical environmental concentrations (CECs) were proposed by Fick et al. (2010) and utilise the concept of the FPM but are independent of environmental concentrations. CECs are calculated by the ratio (Eq. 9) of H_T_PC and P_Blood:Water_ (Eq. 8).

9 $$ \mathrm{CEC}=\frac{{\mathrm{H}}_{\mathrm{T}}\mathrm{PC}}{{\mathrm{P}}_{\mathrm{Blood}:\mathrm{Water}}} $$

#### Risk quotients

Information on the acute toxicity of each of the compounds was obtained from reviews containing comprehensive experimental ecotoxicological data or studies containing such data provided by pharmaceutical companies (Sanderson and Thomsen 2009; Sangion and Gramatica 2016a; Vestel et al. 2016). For compounds not included in these studies, LC50 values were obtained from risk assessments or scientific literature (supplementary material S4). Values were only included if they followed standard protocols (for example, OECD, US EPA), used at least five concentrations in the exposures and at least three replicates per treatment. This data was unavailable for 12 compounds, so ECOSAR (v 1.11) was used to estimate LC_50_ values although the model was unable to estimate these for 7 of the compounds. A relative ranking, where the ranking was divided by the number of compounds in the scheme, was used in order to compare rankings across all effect schemes.

Risk quotients (RQ) were calculated by dividing the lowest LC_50_ value for fish, algae or daphnia by each of the PECs calculated in “Calculations” section. An assessment factor of 1000 was applied in order to account for any uncertainties and provide a more conservative assessment. Those compounds with a RQ > 1 deemed to be hazardous to the environment.

## Results

### Exposure criteria

#### Comparison of predicted environmental concentrations between schemes

**Fig. 1:**
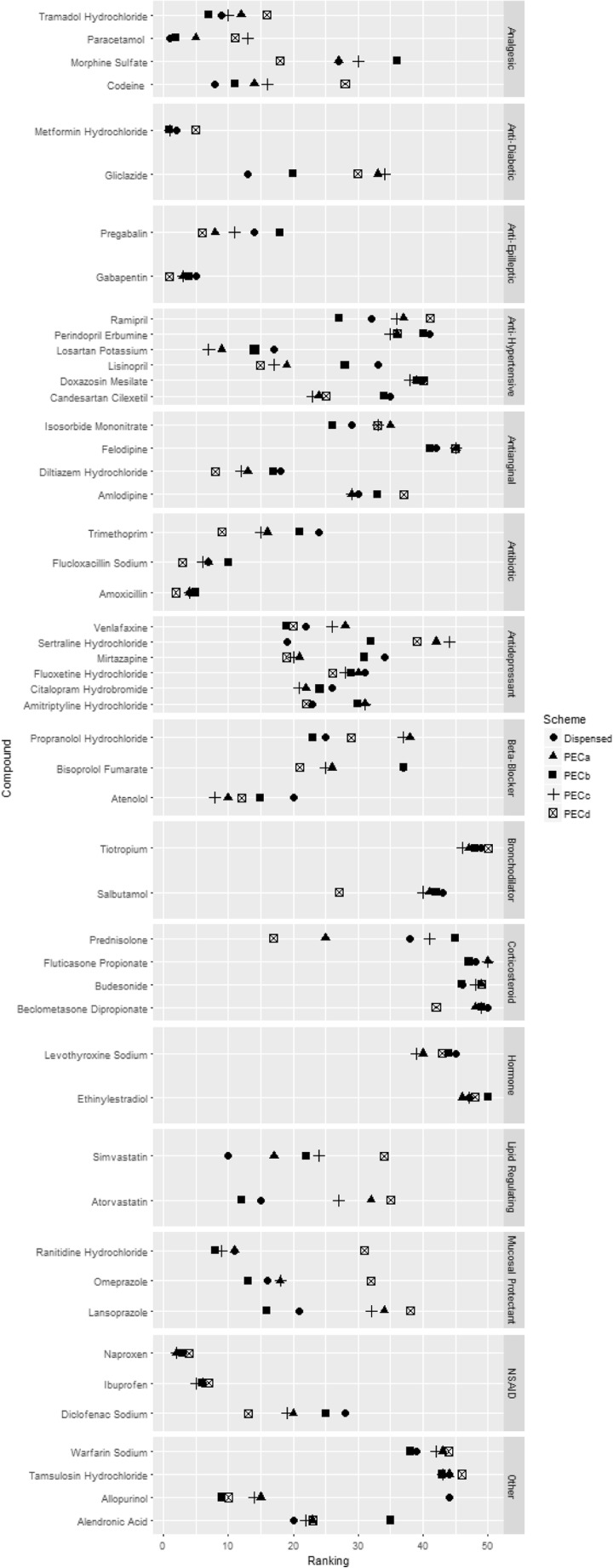
Comparison of ranking of pharmaceuticals by compound class between predicted environmental concentration schemes

Metformin, gabapentin, flucloxacillin, amoxicillin, naproxen and ibuprofen were ranked in the top 10 across all PEC schemes, whereas tamsulosin, ethinylestradiol, fluticasone, budesonide, beclomethasone, felodipine and tiotropium were ranked in the bottom 10 (Fig. 1). These compounds were in the top 10 and bottom 10 respectively when ranked by the amount dispensed annually. For most compounds, there was less than a 10 place difference between schemes (supplementary material S3). Where larger differences occurred, it can mostly be attributed to different results between schemes which utilised usage data (PEC_A_, PEC_B_ and PEC_C_) and PEC_D_ which did not. However, the PEC values for individual compounds did differ greatly depending on which scheme was used.

#### Comparison with measured environmental concentrations

**Fig. 2:**
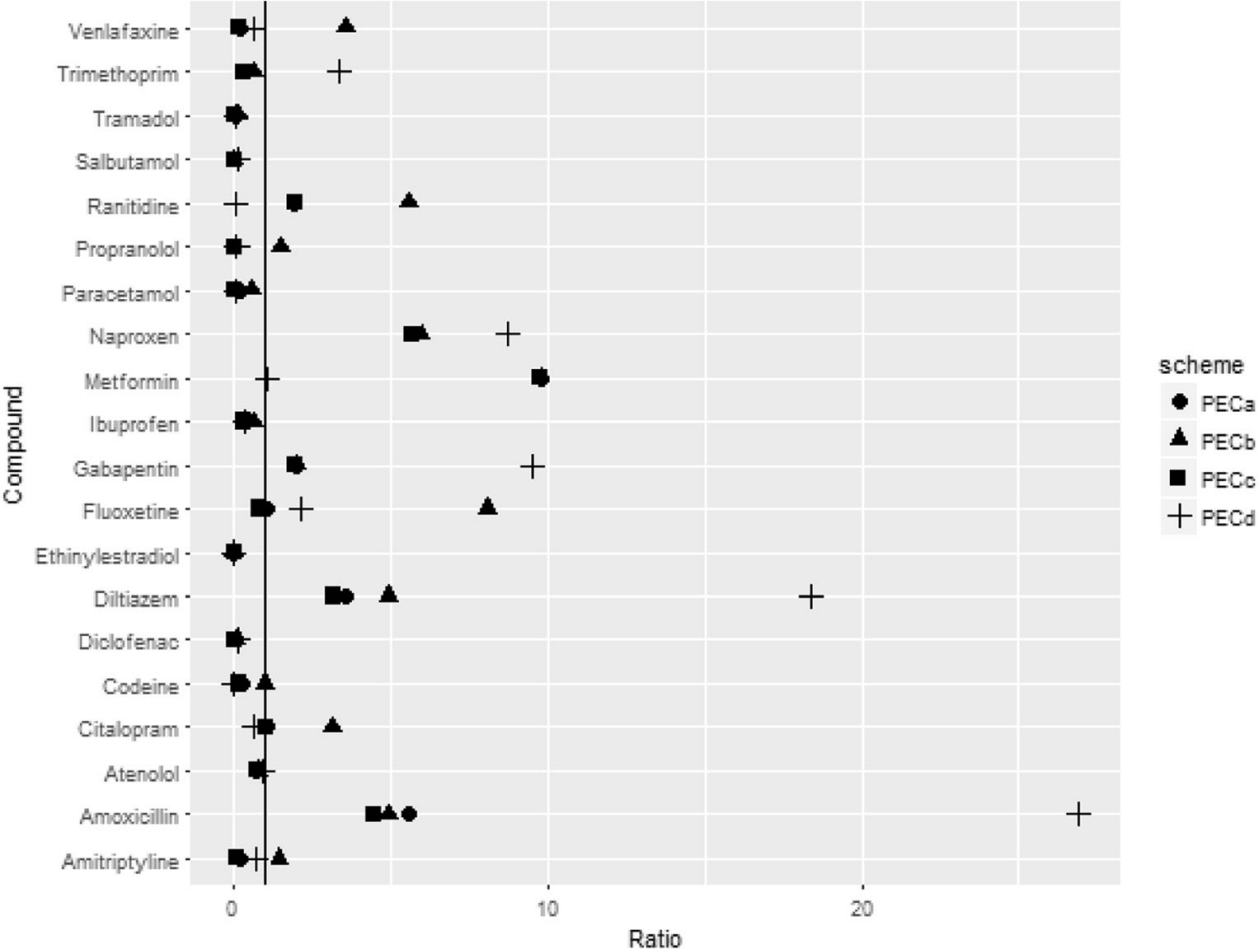
Ratio of PEC: maximum MEC for each of the schemes. The line denotes a ratio of one

MECs in the UK were available for 24 out of the 50 study compounds. Of these, warfarin sodium, sertraline prednisolone and fluticasone propionate were below the limit of detection (LOD) in all studies. All of the schemes underestimated the maximum concentrations for tramadol, salbutamol, paracetamol, ibuprofen and ethinylestradiol (Fig. 2). Maximum MECs were overestimated for amoxicillin, diltiazem, gabapentin and naproxen by all schemes. For the other compounds, PEC_B_ overestimated maximum concentrations more than the other schemes.

**Fig. 3:**
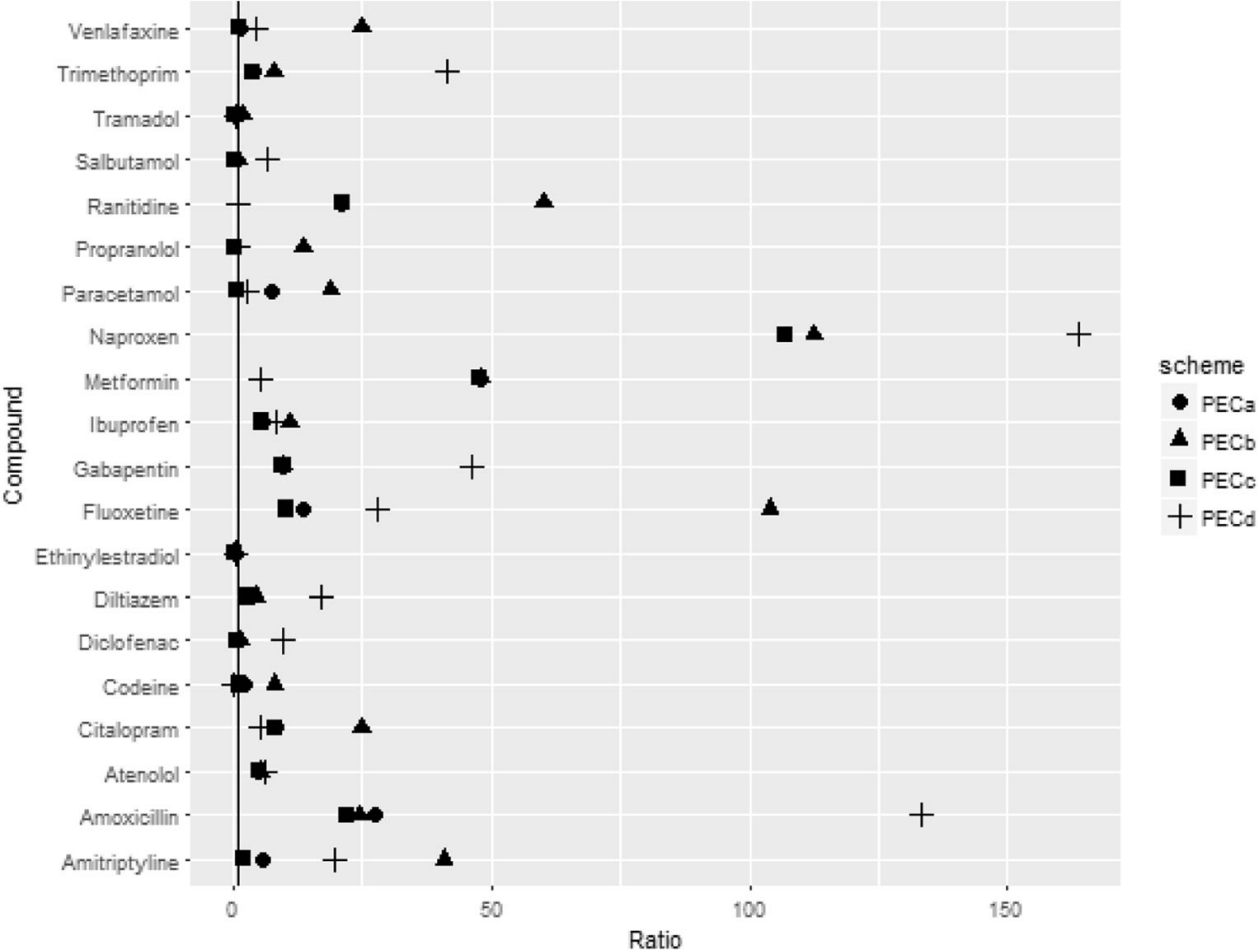
Ratios of PEC: mean MEC for each of the schemes. The line denotes a ratio of one

All PECs were overestimates of mean MECs for all of the compounds, with the exception of ethinylestradiol and salbutamol (Fig. 3). PEC_A_, PEC_C_ and PEC_D_ also underestimated the MECs of propranolol and tramadol. Further to this, PEC_C_ and PEC_D_ underestimated the MECs for paracetamol and codeine, respectively. The ratio for mean MECs was much higher than those for maximum MECs for all compounds. PEC_D_ overestimated MECs to a greater degree than the other schemes, and PEC_C_ more accurately predicted the mean MECs than the other schemes.

### Effect criteria

**Fig. 4:**
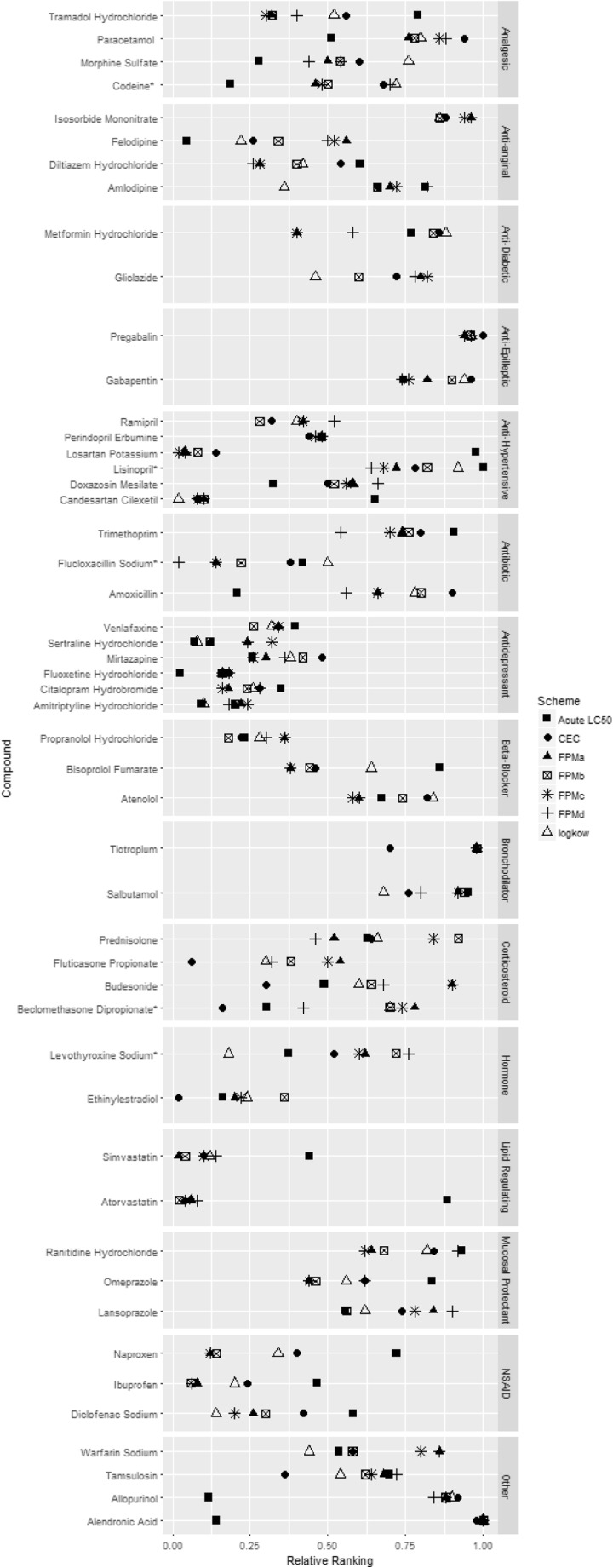
Comparison of the relative ranking of compounds between effect schemes: acute LC_50_, critical environmental concentrations, LOG_KOW_ and fish plasma model using PEC_A_ (FPMa), PEC_B_ (FPMb), PEC_c_ (FPMc) and PEC_D_ (FPMd)

For many of the compounds, LC_50_ values resulted in the opposite ranking to the other schemes (supplementary material S5). The FPM model, LOG_KOW_ and CEC schemes resulted in simvastatin, atorvastatin, candesartan, ibuprofen and losartan being ranked in the top 25%; however, the LC_50_ ranked these compounds as lower priority (Fig. 4). The opposite was true for allopurinol, alendronic acid, beclomethasone and amoxicillin. Pregabalin, gabapentin, isosorbide mononitrate and tiotropium were ranked in the bottom 25% across all schemes. CECs highlighted some compounds as priority that the other schemes did not; ethinylestradiol, fluticasone propionate and beclomethasone diproprionate had a higher relative ranking before the inclusion of PEC values. As a compound class, antidepressants and antibiotics were given a high priority ranking, whereas bronchodilators and mucosal protectants did not.

All compounds had an ER ratio < 1000, with the exception of tiotropium and alendronic acid, where the ER exceeded this value with all PECs (supplementary material S6). Isosorbide mononitrate also had an ER < 1000 for FPM_A_, and FPM_C_. Less compounds exceeded the RQ value of 1; all PECs resulted in an RQ > 1 for amoxicillin (supplementary material S6). PEC_B_ resulted in the RQ being exceeded for the allopurinol and fluoxetine and PEC_D_ for allopurinol, fluoxetine and flucloxacillin.

## Discussion

### Comparison of schemes for predicted environmental concentrations

For many of the compounds in this assessment, the ranking within each PEC scheme was correlated with the amount dispensed, which has also been found in other prioritisation studies (Ashton et al. 2004; Roos et al. 2012). Of the compounds which were ranked in the top 10 across all schemes, metformin, amoxicillin, naproxen and ibuprofen have previously occurred on many priority lists (Burns et al. 2018b). Gabapentin and flucloxacillin have only been listed of concern in one prioritisation exercise each (Helwig et al. 2013; Ortiz de García et al. 2013) and, as a result, monitoring studies including these compounds are much lower. PEC_D_ results were less closely related with the amount of compound dispensed, as this was not included in the calculation. Instead, PEC_D_ used the maximum dosage and assumed 1% of the population was taking the compound. It is unsurprising that compounds which have a higher dosage are also prescribed at higher masses. However, for many compounds, the usage has been found to surpass 1% (Pereira et al. 2017). As a result, the inclusion of usage data in risk assessments is very important and, where this is not available (e.g. many developing countries), its production should be seen as a high priority by governments. As over the counter (OTC) sales of some products have been attributed to up to 50% of this, it is very important that these figures are available for risk assessment purposes (Guo et al. 2016). Of the compounds assessed in the current study, paracetamol, ibuprofen, diclofenac, omeprazole and naproxen are available OTC in the UK. Even though OTC data were not available, omeprazole was ranked between 10 and 20 across all schemes, and if OTC sales were also included, it could be much more important in terms of environmental impact. Furthermore, many pharmaceuticals are also used for veterinary purposes and these data are needed for more accurate PEC production.

Although, for the majority of compounds, ranking by the amount of pharmaceutical dispensed may be sufficient to estimate relative environmental exposure, some compounds undergo extensive metabolism or removal in WWTPs, making some refinement necessary. Amoxicillin, metformin, gabapentin, ibuprofen and naproxen are prescribed in such high numbers that the application of removal and excretion data has little impact on their relative ranking. Gliclazide, on the other hand, had a 20 place ranking difference between the amount dispensed and PEC_A_ due to its extensive metabolism. Those which were ranked between 20 and 40 showed more variability in their ranking between schemes than those at the top and bottom end, as they were dispensed in similar amount to other compounds. Information on the metabolism of pharmaceuticals was available in the scientific literature and pharmaceutical databases, with little variation in reported values.

Removal rates during wastewater treatment had less of an effect on the ranking of compounds than excretion rate. It is possible that this is the result of the overall lack of information of this process or variability within the data, depending on external factors such as temperature and WWTP efficiency (Golovko et al. 2014). For example, removal of metformin has been reported to be as low as 0% and as high as 99% (Santos et al. 2013). Variability such as this can have a great impact on the ranking of compounds; PEC_B_ included the lower rate of removal of 0% which resulted in a ranking of 1; however, using the higher removal rate of 99% would have resulted in the lower ranking of 23. Furthermore, in some cases, an increase in the compound concentration has been seen in effluent as the result of conversion back to the parent compound in WWTPs and so a negative removal rate would have to be used in a PEC scheme to accurately take this occurrence into account (Paíga et al. 2016).

### Comparison of predicted environmental concentrations with measured environmental concentrations

In the majority of cases, the PECs failed to accurately represent the MECs; mean MECs were mostly overestimated, and half of the maximum MECs were underestimated by all schemes. PEC_A_, PEC_B_ and PEC_C_ were most accurate in estimating mean MECs, despite overestimations. Nevertheless, these afford a degree of environmental safety. When interpreting these results, the lack of available monitoring data needs to be taken into consideration and many compounds were only measured at one time point and at one or two sites. Concentrations of some pharmaceuticals have been shown to fluctuate depending on seasonal and environmental conditions, so more thorough monitoring studies are needed to further validate methods for producing PECs (Moreno-González et al. 2015). Ferrari et al. (2004) compared PEC_B_ and the highest MECs for five pharmaceuticals in wastewater effluent and rivers in France and Germany. In German effluents, these concentrations were accurately predicted for carbamazepine and diclofenac, but were underestimated (although by less than a factor of 10) for propranolol, clofibric acid and sulfamethoxazole, and overestimated for oflaxin. However, in French effluents, MECs were overestimated for all compounds showing that the scenario being assessed is important when choosing a PEC model and that local factors which could affect concentrations are considered. Burns et al. (2017) also compared MECs and PECs which were calculated using local hydrological information alongside lowest removal and highest excretion rates. MECs were accurately predicted in one river but not another, which was attributed to missing inputs. The inclusion of local hydrological information such as this may help to produce more accurate PECs.

### Comparison of effect-based methods

FPM, Log_KOW_ and CEC schemes resulted in different rankings to acute LC_50_ and triggered different compounds for further assessment, which is concurrent with other recent studies, showing that Log_KOW_ has a strong influence on these calculations (Roos et al. 2012). Additionally, FPMs were more conservative than RQs, triggering more compounds for further assessment. Thus, simply ranking compounds by log_KOW_ could be a useful approach for determining the relative hazard pharmaceuticals pose to biota. Nevertheless, although log_KOW_ is used in FPM and CEC models, it does not necessarily indicate the compound will be toxic, but instead that it is likely to be taken up by fish at a level sufficient to have a biological effect (Schrieber et al. 2011). Instead, it is suggested that those with an ER less than 1000 warrant further assessment (Huggett et al. 2003). Log_KOW_ values have been used as predictors for bioconcentration; however, this measurement was originally developed for non-polar chemicals, and as a result does not work for many chemicals (Schrieber et al. 2011).

The use of acute LC_50_ and QSAR in order to assess the potential hazard of pharmaceuticals has been debated. Although LC_50_ values are derived from experimental work, they can be influenced by variables such as the number of concentrations assessed (Hoyett et al. 2016). The primary concern relating to pharmaceuticals in the environment is the potential chronic exposure to low levels, and not acute toxicity. As a result, they may affect endpoints which are not covered by traditional risk assessments (Johnson et al. 2017). QSARs have been used to model the potential toxicity of contaminants to fish, daphnia and algae. There are several QSAR models which have been proposed for use in predicting ecotoxicity of pharmaceuticals which have found to vary in accuracy (for example, de Roode et al. 2006; Sangion and Gramatica 2016a).

There is evidence that fish are more sensitive than algae or invertebrates as they retain many of the same drug targets as humans (Donnachie et al. 2016). The FPM was developed in order to utilise this information. A read-across approach can be used in assessing the potential risk of pharmaceuticals to invertebrates and algae. Fish share 86% of targets with humans, 61% have been found to be conserved in daphnia and 35% in algae (Gunnarsson et al. 2008). There is particular concern surrounding the toxicity of antibiotics and statins to algae, in part due to conserved pathways, but also due to the inhibition of symbiotic bacteria (Guo et al. 2015). CEC resulted in a higher ranking for statins and two of the antibiotics than LC_50_ values. Amoxicillin, on the other hand, was highlighted by its acute toxicity and not by the FPM. Only the RQ which included PEC_A_ exceeded 1 for amoxicillin, whereas this was exceeded by all of the FPM schemes. As a result, the FPM and CEC will add a degree of protection for organisms besides fish.

For many compounds, FPM and CECs resulted in similar rankings. The minor influence PEC has on FPM confirms what has been found in other comparisons between prioritisation schemes (Roos et al. 2012). However, ethinylestradiol, fluticasone propionate and beclomethasone were highlighted by CECs, but not by FPMs as the PEC values for these compounds were small. In this case, ethinylestradiol had a low PEC; however, MECs were much higher than this. Ethinylestradiol is a compound on the EU’s priority watch list due to concern over its potential effects at environmentally relevant concentrations. Johnson et al. (2017) ranked chemicals based on their measured environmental concentrations in UK rivers and measured ecotoxicity concentrations, and found that ethinylestradiol was highlighted as posing the greatest risk. As a result, it is important that PEC results are accurate if FPM is going to be used. The use of an assessment factor or ER value of 1000 allows for the most conservative estimate of risk whilst accounting for uncertainty in the PEC values.

### Selecting a prioritisation scheme

It is important to consider the inclusion of compounds into a scheme to begin with. Metoprolol, carbamazepine, aspirin and sulfamethoxazole were four of the most cited pharmaceuticals of concern in the prioritisation literature but were not in the 50 most prescribed compounds (Donnachie et al. 2016). The high number of prescriptions does not necessarily translate into a large mass of the compound; bronchodilators, for example, were prescribed in high numbers, but at a very low mass. As a result, certain compounds may be overlooked and it may be necessary to select compounds based on their mass as well as prescription numbers.

Of the PEC schemes used in this assessment, PEC_A_ is the most suitable for assessing the relative exposure risk as it requires limited data, but also conservatively estimates the likelihood of pharmaceuticals entering the environment. It can be used to select pharmaceuticals for which to further refine PECs based on local criteria before selection of compounds for monitoring in the environment. Where information on the number of prescriptions is not available, PEC_D_ is a better alternative as it can work within the confines of available data.

Assessment of the potential effects of pharmaceuticals should be used alongside PEC evaluations. Log_KOW_ offers a quick and easy method for assessing the relative risk, based on potential bioaccumulation. The use of CECs and FPM add an extra level of refinement, based on utilising information on mammalian effects. FPM appears to give a conservative approach to prioritising pharmaceuticals in comparison to acute RQs. As a result, those compounds which also exceed the RQ threshold should be of greater priority. The use of CECs over FPMs allows the ranking of compounds independent of PECs. However, both exposure risk and potential effects should be included, as compounds found at small concentrations could still be enough to warrant an effect. For example, ethinylestradiol was ranked as a low priority by the PEC schemes, but inclusion of effect information increased its ranking.

When prioritising pharmaceuticals, it is essential to take a holistic approach which conservatively highlights potential compounds of concern which warrant further assessment. It is important to consider why the exercise is being carried out and the question it is trying to address. There will not be a one size fits all approach, and not all schemes will be appropriate in all situations. As a result, the limitations to each of these schemes need to be kept in mind.

### Compounds of concern

The combination of PEC and effect criteria clearly highlights groups which should be a priority for further research. Some assessments have only added one compound from each class to the priority list, assuming that each class will have a similar mode of action and similar effect (Besse and Garric 2008). Antidepressants were ranked high across all of the effect schemes, and moderately for PECs too. Overall ranking between compounds does not differ much; however, fluoxetine may be of most of concern due to exceeding the RQ threshold values when none of the others did. Fluoxetine is commonly present on priority lists; however, some rankings have pointed towards sertraline, citalopram and amitriptyline as representing a greater hazard (Besse and Garric 2008; Roos et al. 2012; Sangion and Gramatica 2016b). Many of these antidepressants have been found to have an effect on biota at environmentally relevant concentrations and the use of FPM also highlights this (Silva et al. 2015). To the authors’ knowledge, this is the first prioritisation exercise which has highlighted mirtazapine and venlafaxine to be a potential concern.

Similarly to antidepressants, candesartan and losartan had moderate PEC rankings but high effect rankings for FPM, CEC and LC_50_, whilst other anti-hypertensives had a low ranking across both PEC and effect schemes. These compounds are not commonly included in prioritisation exercises; however, losartan has been present on priority lists previously (Besse and Garric 2008). Candesartan had a higher ranking across schemes and as a result may be more of a concern. The lipid regulators, atorvastatin and simvastatin also had moderate to low PECs. However, their high ranking among CECs and FPM means they warrant further investigation.

Amoxicillin and flucloxacillin were two of four compounds to exceed a RQ value of 1. Both of these compounds were ranked highly as the result of PEC values. The effect rankings of flucloxacillin were much higher than those of amoxicillin. Flucloxacillin is not commonly present in monitoring or effects studies and there is still uncertainty about its occurrence and impacts so it could be seen as a priority compound.

Ibuprofen was ranked in the top 10 of all of schemes, with the exception of acute LC_50_. Ibuprofen is the fifth most prioritised compound in the prioritisation literature (Burns et al. 2018b). The environmental impact of ibuprofen pollution has been the focus of many studies and its repeat presence on priority lists and high rankings in the current study indicate the importance in understanding its fate and effects.

Allopurinol may also warrant further assessment due to its high exposure ranking and RQ value. Whilst it had a low ranking for FPM, CEC and Log_KOW_ values, it had an ER < 1000. Although Allopurinol has been stated to be a highly prescribed drug in other EU countries (Küster and Adler 2014; Roos et al. 2012), Roos et al. (2012) carried out a comparison of first-tier prioritisation schemes, including FPM, on 582 pharmaceuticals in Sweden, and did not find it to be a high priority. However, it has been highlighted on other priority lists based on exposure and effect criteria (Besse and Garric 2008; Linert et al. 2007). Despite this, it is not present in the monitoring or ecotoxicity literature and it has only been monitored in coastal waters in Spain, where it was not detected (Rodrígues-Navas et al. 2013).

Other compounds such as metformin and gabapentin are ranked in the top by PEC schemes, but inclusion of effect criteria decreased their ranking. However, due their high PECs, moderate effect rankings across FPM and acute LC_50_ values, they may still warrant further assessment. It is particularly important to understand their occurrence and fate. Metformin in particular may be of concern as it now a widely used drug, and its usage has increased rapidly over the last decade (Oosterhuis et al. 2013).

This assessment also clearly highlights compounds which are not of concern. Bronchodilators were ranked in the bottom of all schemes and corticosteroids were ranked at the bottom across all PEC schemes. This is concurrent with other prioritisation exercises. As a result, these compounds are not commonly featured in monitoring campaigns or experimental effects work. Although the priority ranking of corticosteroids increased with the application of effect criteria, it was still low.

### Future direction for the management of pharmaceuticals in the environment

There is some evidence that EU policy has not used risk assessment approaches to accurately identify compounds of concern. In the present study, ibuprofen and naproxen had a higher PEC and effect ranking than diclofenac even though the latter has been placed on the EU priority watch list. This could perhaps be attributed to the fall in diclofenac’s usage over the past few years though (Mavragani et al. 2016). Ethinylestradiol is another compound included on the EU priority watch list even though it had a low PEC ranking and similar effect ranking; only CECs ranked it as a priority. Similar results were seen in comparison of first-tier risk assessments by Roos et al. (2012), where FPM did not result in a high ranking for ethinylestradiol but CEC and three other schemes did. As pharmaceuticals are designed to be biologically active, it is important that there is an understanding of these pathways in non-target organisms in order to create better risk assessments.

There has been an increasing interest in the occurrence of pharmaceuticals in environmental compartments other than effluent and water such as sediment and marine environments. Comparatively, little is known about the occurrence of pharmaceuticals in these areas (Fabbri and Franzellitti 2016; Gaw et al. 2014) and use of the PEC schemes employed here may not appropriately predict presence in these compartments. Other properties, such as lipophilicity, pH and sediment type may be more relevant in predicting the presence of pharmaceuticals in sediments, and in turn the potential risks to biota which live within these systems (Al-Khazrajy and Boxall 2016). Salinity is also a defining factor of marine waters and it is hypothesised that the physical-chemical characteristics of some compounds may change in marine waters. For example, the partition coefficient between sediment and water for estrone increases with increasing salinity, meaning concentrations will be lower (Pal et al. 2010).

All pharmaceuticals are metabolised to a different degree, yet only two prioritisation schemes have included metabolites (Besse and Garric 2008; Capleton et al. 2006). If metabolism and degradation play a significant role in the fate of pharmaceuticals then metabolites will be present in the environment. Few studies have covered the occurrence and effects of metabolites, many of which are inert, but some of which have been found to be pharmacologically active and even toxic (García-Cambero et al. 2015).

## Conclusion

Prioritisation schemes should include assessments of the potential of a compound to enter the environment as well its potential toxicity. Excretion of pharmaceuticals had a large influence on the ranking of PECs for different compounds, and as a result should be included in these calculations. CECs should be used alongside PECs in order to assess potential hazard; both of these schemes result in a conservative estimate of risk, and highlight compounds which warrant further assessment. Antidepressants, statins, antibiotics, candesartan, losartan and ibuprofen were highlighted as the substances of greatest environmental concern.

## Electronic supplementary material

ESM 1 (XLSX 49 kb)

## References

[CR1] Adler NE, Koschorreck J, Rechenberg B (2008). Environmental impact assessment and control of pharmaceuticals: the role of environmental agencies. Water Sci Technol.

[CR2] Al-Khazrajy OSA, Boxall ABA (2016). Impacts of compound properties and sediment characteristics on the sorption behaviour of pharmaceuticals in aquatic systems. J Hazard Mater.

[CR3] Ashton D, Hilton M, Thomas KV (2004). Investigating the environmental transport of human pharmaceuticals to streams in the United Kingdom. Sci Total Environ.

[CR4] Backhaus T, Faust M (2012). Predictive environmental risk assessment of chemical mixtures: a conceptual framework. Environ Sci Technol.

[CR5] Baker DR, Kasprzyk-Hordern B (2013). Spatial and temporal occurrence of pharmaceuticals and illicit drugs in the aqueous environment and during wastewater treatment: new developments. Sci Total Environ.

[CR6] Besse JP, Garric J (2008). Human pharmaceuticals in surface waters. Implementation of a prioritization methodology and application to the French situation. Toxicol Lett.

[CR7] Besse JP, Kausch-Barreto C, Garric J (2008). Exposure assessment of pharmaceuticals and their metabolites in the aquatic environment: application to the French situation and preliminary prioritization. Hum Ecol Risk Assess.

[CR8] Bound JP, Voulvoulis N (2006). Predicted and measured concentrations for selected pharmaceuticals in UK rivers: implications for risk assessment. Water Res.

[CR9] Burns EE, Thomas-Oates J, Kolpin DW, Furlong ET, Boxall AB (2017). Are exposure predictions, used for the prioritization of pharmaceuticals in the environment, fit for purpose?. Environ Toxicol Chem.

[CR10] Burns EE, Carter LJ, Kolpin DW, Thomas-Oates J, Boxall ABA (2018). Temporal and spatial variation in pharmaceutical concentrations in an urban river system. Water Res.

[CR11] Burns EE, Carter LJ, Snape JR, Thomas-Oates J, Boxall ABA (2018). Application of prioritization approaches to optimize environmental monitoring and testing of pharmaceuticals. J Toxicol Environ Health B Crit Rev.

[CR12] Capleton AC, Courage C, Rumsby P, Holmes P, Stutt E, Boxall ABA, Levy LS (2006). Prioritising veterinary medicines according to their potential indirect human exposure and toxicity profile. Toxicol Lett.

[CR13] Dong Z, Senn DB, Moran RE, Shine JP (2013). Prioritizing environmental risk of prescription pharmaceuticals. Regul Toxicol Pharmacol.

[CR14] Donnachie RL, Johnson AC, Sumpter JP (2016). A rational approach to selecting and ranking some pharmaceuticals of concern for the aquatic environment and their relative importance compared with other chemicals. Environ Toxicol Chem.

[CR15] EMEA (2006) Guideline on the environmental risk assessment of medicinal products for human use. Available from: http://www.ema.europa.eu/docs/en_GB/document_library/Scientific_guideline/2009/10/WC500003978.pdf. Accessed 17 May 2018

[CR16] EU (2003) Environmental risk assessment for human medicinal products containing or consisting of GMOs, III/5504/94 draft 4. Available from: https://echa.europa.eu/documents/10162/16960216/tgdpart2_2ed_en.pdf. Accessed 17 May 2018

[CR17] Fabbri E, Franzellitti S (2016). Human pharmaceuticals in the marine environment: focus on exposure and biological effects in animal species. Environ Toxicol Chem.

[CR18] Ferrari B, Mons R, Vollat B, Fraysse B, Paxéus N, Lo Giudice R, Pollio A, Garric J (2004). Environmental risk assessment of six human pharmaceuticals: are the current environmental risk assessment procedures sufficient for the protection of the aquatic environment?. Environ Toxicol Chem.

[CR19] Fick J, Lindberg RH, Tysklind M, Larsson DGJ (2010). Predicted critical environmental concentrations for 500 pharmaceuticals. Regul Toxicol Pharmacol.

[CR20] García-Cambero J, García-Cortés H, Valcárcel Y, Catalá M (2015). Environmental concentrations of the cocaine metabolite benzoylecgonine induced sublethal toxicity in the development of plants but not in a zebrafish embryo–larval model. J Hazard Mater.

[CR21] Gaw S, Thomas KV, Hutchinson TH (2014). Sources, impacts and trends of pharmaceuticals in the marine and coastal environment. Philos Trans R Soc Lond Ser B Biol Sci.

[CR22] Golovko O, Kumar V, Fedorova G, Randak T, Grabic R (2014). Seasonal changes in antibiotics, antidepressants/psychiatric drugs, antihistamines and lipid regulators in a wastewater treatment plant. Chemosphere.

[CR23] Gunnarsson L, Jauhiainen A, Kristiansson E, Nerman O, Larsson DGJ (2008) Evolutionary conservation of human drug targets in organisms used for environmental risk assessments. Environ Sci Technol 42(15):5807–5813 10.1021/es8005173 18754513

[CR24] Guo J, Boxall ABA, Selby K (2015). Do pharmaceuticals pose a threat to primary producers?. Crit Rev Environ Sci Technol.

[CR25] Guo J, Sinclair CJ, Selby K, Boxall ABA (2016). Toxicological and ecotoxicological risk-based prioritization of pharmaceuticals in the natural environment. Environ Toxicol Chem.

[CR26] Helwig K, Hunter C, McNaughtan M, Roberts J, Pahl O (2013). Ranking prescribed pharmaceuticals in terms of environmental risk: inclusion of hospital data and the importance of regular review. Environ Toxicol Chem.

[CR27] Hoyett Z, Owens MA, Clark CJ, Abazinge M (2016). A comparative evaluation of environmental risk assessment strategies for pharmaceuticals and personal care products. Ocean Coast Manag.

[CR28] Huggett DB, Cook JC, Ericson JF, Williams RT (2003). A theoretical model for utilizing mammalian pharmacology and safety data to prioritize potential impacts of human pharmaceuticals to fish. Hum Ecol Risk Assess.

[CR29] Hughes SR, Kay P, Brown LE (2013). Global synthesis and critical evaluation of pharmaceutical data sets collected from river systems. Environ Sci Technol.

[CR30] Johnson AC, Donnachie RL, Sumpter JP, Jürgens MD, Moeckel C, Pereira MG (2017). An alternative approach to risk rank chemicals on the threat they pose to the aquatic environment. Sci Total Environ.

[CR31] Kasprzyk-Hordern B, Dinsdale RM, Guwy AJ (2008). The occurrence of pharmaceuticals, personal care products, endocrine disruptors and illicit drugs in surface water in South Wales, UK. Water Res.

[CR32] Kasprzyk-Hordern B, Dinsdale RM, Guwy AJ (2009). The removal of pharmaceuticals, personal care products, endocrine disruptors and illicit drugs during wastewater treatment and its impact on the quality of receiving waters. Water Res.

[CR33] Kay P, Hughes SR, Ault JR, Ashcroft AE, Brown LE (2016). Widespread, routine occurrence of pharmaceuticals in sewage effluent, combined sewer overflows and receiving waters. Environ Pollut.

[CR34] Kostich MS, Lazorchak JM (2008). Risks to aquatic organisms posed by human pharmaceutical use. Sci Total Environ.

[CR35] Küster A, Adler N (2014). Pharmaceuticals in the environment: scientific evidence of risks and its regulation. Philos Trans R Soc Lond Ser B Biol Sci.

[CR36] LaLone CA, Berninger JP, Villeneuve DL, Ankley GT (2014). Leveraging existing data for prioritization of the ecological risks of human and veterinary pharmaceuticals to aquatic organisms. Philos Trans R Soc Lond Ser B Biol Sci.

[CR37] Linert J, Güdel K, Escher BI (2007). Screening method for ecotoxicological hazard assessment of 42 pharmaceuticals considering human metabolism and excretory routes. Environ Sci Technol.

[CR38] Mansour F, Al-Hindi M, Saad W, Salam D (2016). Environmental risk analysis and prioritization of pharmaceuticals in a developing world context. Sci Total Environ.

[CR39] Mavragani A, Sypsa K, Sampri A, Tsagarakis K (2016). Quantifying the UK online interest in substances of the EU watchlist for water monitoring: diclofenac, estradiol, and the macrolide antibiotics. Water.

[CR40] Moreno-González R, Rodriguez-Mozaz S, Gros M, Barceló D, León VM (2015). Seasonal distribution of pharmaceuticals in marine water and sediment from a Mediterranean coastal lagoon (SE Spain). Environ Res.

[CR41] Nakada N, Hanamoto S, Jürgens MD, Johnson AC, Bowes MJ, Tanaka H (2017). Assessing the population equivalent and performance of wastewater treatment through the ratios of pharmaceuticals and personal care products present in a river basin: application to the River Thames basin, UK. Sci Total Environ.

[CR42] NHS (2014) GP practice prescribing presentation-level data. Available from: http://content.digital.nhs.uk

[CR43] Oosterhuis M, Sacher F, ter Laak TL (2013). Prediction of concentration levels of metformin and other high consumption pharmaceuticals in wastewater and regional surface water based on sales data. Sci Total Environ.

[CR44] Ortiz de García S, Pinto GP, García-Encina PA, Mata RI (2013). Ranking of concern, based on environmental indexes, for pharmaceutical and personal care products: an application to the Spanish case. J Environ Manag.

[CR45] Paíga P, Santos LHMLM, Ramos S, Jorge S, Silva JG, Delerue-Matos C (2016). Presence of pharmaceuticals in the Lis River (Portugal): sources, fate and seasonal variation. Sci Total Environ.

[CR46] Pal A, Gin KYH, Lin AYC, Reinhard M (2010). Impacts of emerging organic contaminants on freshwater resources: review of recent occurrences, sources, fate and effects. Sci Total Environ.

[CR47] Pereira AMPT, Silva LJG, Lino CM, Meisel LM, Pena A (2017). A critical evaluation of different parameters for estimating pharmaceutical exposure seeking an improved environmental risk assessment. Sci Total Environ.

[CR48] Roberts PH, Thomas KV (2006). The occurrence of selected pharmaceuticals in wastewater effluent and surface waters of the lower Tyne catchment. Sci Total Environ.

[CR49] Rodrígues-Navas C, Björklund E, Bak SA, Hansen M, Krogh KA, Maya F, Forteza R, Cerdà V (2013). Pollution pathways of pharmaceutical residues in the aquatic environment on the Island of Mallorca, Spain. Arch Environ Contam Toxicol.

[CR50] de Roode D, Hoekzema C, de Vries-Buitenweg S, van de Waart B, van der Hoeven J (2006). QSARs in ecotoxicological risk assessment. Regul Toxicol Pharmacol.

[CR51] Roos V, Gunnarsson L, Fick J, Larsson DGJ, Rudén C (2012). Prioritising pharmaceuticals for environmental risk assessment: towards adequate and feasible first-tier selection. Sci Total Environ.

[CR52] Sanderson H, Thomsen M (2009). Comparative analysis of pharmaceuticals versus industrial chemicals acute aquatic toxicity classification according to the United Nations classification system for chemicals. Assessment of the (Q)SAR predictability of pharmaceuticals acute aquatic toxicity. Toxicol Lett.

[CR53] Sanderson H, Johnson DJ, Reitsma T, Brain RA, Wilson CJ, Solomon KR (2004). Ranking and prioritization of environmental risks of pharmaceuticals in surface waters. Regul Toxicol Pharmacol.

[CR54] Sangion A, Gramatica P (2016). Hazard of pharmaceuticals for aquatic environment: prioritization by structural approaches and prediction of ecotoxicity. Environ Int.

[CR55] Sangion A, Gramatica P (2016). PBT assessment and prioritisation of contaminants of emerging concern: pharmaceuticals. Environ Res.

[CR56] Santos LH, Gros M, Rodriguez-Mozaz S, Delerue-Matos C, Pena A, Barceló D, Montenegro MC (2013). Contribution of hospital effluents to the load of pharmaceuticals in urban wastewaters: identification of ecologically relevant pharmaceuticals. Sci Total Environ.

[CR57] Schrieber R, Gundel U, Franz S, Kuster A, Rechenberg B, Attenburger R (2011). Using the fish plasma model for comparative hazard identification for pharmaceuticals in the environment by extrapolation from human therapeutic data. Regul Toxicol Pharmacol.

[CR58] Silva LJG, Pereira AMPT, Meisel LM, Lino CM, Pena A (2015). Reviewing the serotonin reuptake inhibitors (SSRIs) footprint in the aquatic biota: uptake, bioaccumulation and ecotoxicology. Environ Pollut.

[CR59] Vestel J, Caldwell DJ, Constantine L, D’Aco VJ, Davidson T, Dolan DG, Millard SP, Murray-Smith R, Parke NJ, Ryan JJ, Straub JO, Wilson P (2016). Use of acute and chronic ecotoxicity data in environmental risk assessment of pharmaceuticals. Environ Toxicol Chem.

